# *Bacteroides uniformis* CECT 7771 requires adaptive immunity to improve glucose tolerance but not to prevent body weight gain in diet-induced obese mice

**DOI:** 10.1186/s40168-024-01810-3

**Published:** 2024-06-06

**Authors:** Marina Romaní-Pérez, Inmaculada López-Almela, Clara Bullich-Vilarrubias, Zoran Evtoski, Alfonso Benítez-Páez, Yolanda Sanz

**Affiliations:** 1 Institute of Agrochemistry and Food Technology, Spanish National Research Council (IATA-CSIC), Paterna-Valencia, 46980 Valencia, Spain; 2 Present Address: Research Group Intracellular Pathogens: Biology and Infection, Department of Animal Production and Health, Veterinary Public Health and Food Science and Technology, Faculty of Veterinary Medicine, Cardenal Herrera-CEU University, Valencia, Spain

**Keywords:** Obesity, Oral glucose intolerance, Gut microbiota, *Bacteroides uniformis*, Intestinal immunity

## Abstract

**Background:**

The metabolic disturbances of obesity can be mitigated by strategies modulating the gut microbiota. In this study, we sought to identify whether innate or adaptive immunity mediates the beneficial metabolic effects of the human intestinal bacterium *Bacteroides uniformis* CECT 7771 in obesity.

**Methods:**

We evaluated the effects of orally administered *B. uniformis* on energy homeostasis, intestinal immunity, hormone levels, and gut microbiota in wild-type and *Rag1*-deficient mice with diet-induced obesity. We also assessed whether *B. uniformis* needed to be viable to exert its beneficial effects in obesity and to directly induce immunoregulatory effects.

**Results:**

The administration of *B. uniformis* to obese mice improved glucose tolerance and insulin secretion, restored the caloric intake suppression after an oral glucose challenge, and reduced hyperglycemia. The pre- and post-prandial glucose-related benefits were associated with restoration of the anti-inflammatory tone mediated by type 2 macrophages and regulatory T cells (Tregs) in the lamina propria of the small intestine. Contrastingly, *B. uniformis* administration failed to improve glucose tolerance in obese *Rag1*^*-/-*^ mice, but prevented the increased body weight gain and adiposity. Overall, the beneficial effects seemed to be independent of enteroendocrine effects and of major changes in gut microbiota composition. *B. uniformis* directly induced Tregs generation from naïve CD4+ T cells in vitro and was not required to be viable to improve glucose homeostasis but its viability was necessary to prevent body weight gain in diet-induced obese wild-type mice.

**Conclusions:**

Here we demonstrate that *B. uniformis* modulates the energy homeostasis in diet-induced obese mice through different mechanisms. The bacterium improves oral glucose tolerance by adaptive immunity-dependent mechanisms that do not require cell viability and prevents body weight gain by adaptive immunity-independent mechanisms which require cell viability.

Video Abstract

**Supplementary Information:**

The online version contains supplementary material available at 10.1186/s40168-024-01810-3.

## Introduction

The prevalence of obesity and its comorbidities, such as cardiovascular diseases and type 2 diabetes, has shown an alarming increase in the last three decades, reflecting an unmet need for more effective measures to tackle this condition and its complications.

The intake of diets high in fats and simple sugars (so-called Western diets) in combination with a sedentary lifestyle is the principal cause of obesity. The increase in fat depots in obesity, particularly visceral adipose tissue, associates with macrophage-induced inflammation, which perturbs insulin sensitivity [1, 2]. Adipose tissue inflammation correlates with low-grade systemic inflammation that impairs the functionality of other tissues and organs, leading to the dysregulation of energy metabolism and contributing to comorbidities and mortality [3].

Systemic anti-inflammatory therapies have been investigated to treat obesity-associated comorbidities, but they frequently produce undesirable side-effects. More specific strategies targeting key inflammatory mediators have recently been examined [4]; for instance, the administration of nanoparticles delivering the anti-inflammatory agent dexamethasone to adipose-tissue macrophages improves the metabolic phenotype of obese mice [5].

The gut microbiota is acknowledged as a determinant of host metabolism, as its transfer from obese or lean individuals is sufficient to replicate the donor’s phenotype in recipients [6, 7]. Gut microbiota composition is shaped by diet and, specifically, Western diets are associated with reduced microbial richness or diversity [8, 9]. Western diet-induced changes to microbiota composition often contribute to weaken the primary gut defensive mechanisms, reducing mucus thickness [10] and antimicrobial peptide production [11], and can trigger the activation of epithelial and professional immune cells that fuel inflammation [12]. These changes can increase gut permeability and facilitate the translocation of bacterial products such as lipopolysaccharide (LPS) to circulation, inducing metabolic endotoxemia, which spreads inflammation to metabolic organs and causes insulin resistance [13].

The use of drugs and dietary components with anti-inflammatory effects in the gut may counteract inflammation in obesity and, thus, restore metabolic functions. For example, administration of 5-aminosalicylic acid to high-fat diet-fed mice improves glucose homeostasis and microbiota diversity and suppresses inflammation in bowel lamina propria [14]. Likewise, fiber-rich diets and administration of specific intestinal bacterial strains represent novel intervention strategies to improve host energy metabolism through direct or indirect modulation of the intestinal inflammatory tone [15, 16].

We previously showed that the intestinal bacterium *Bacteroides uniformis* CECT 7771 (*B. uniformis*) prevents body weight gain, adiposity, and associated metabolic alterations when administered to obese mice on high-fat diet [17]. Compared with other *B. uniformis* strains, the genome of this bacterium is enriched in glycosyl transferase genes, which in vitro act as mucin-degraders and, in vivo, could theoretically increase the interactions between *B. uniformis* and host intestinal cells [18] and facilitate activation of neuroendocrine and/or immune pathways that control energy metabolism. Indeed, *B. uniformis* restores the obesity-induced increase of the type 1 macrophage (M1)/type 2 (M2) ratio and the reduced abundance of regulatory T cells (Tregs) in Peyer’s patches, immune inductive intestinal sites, and adipose tissue [19]. Nevertheless, a deeper understanding of the mode of action of *B. uniformis* CECT 7771 in obesity is key to advance the development of more efficacious and precision microbiome-based therapies [20] to target specific obesity phenotypes.

Using wild-type (wt) and *Rag1*-deficient (*Rag1*^*-/-*^) mice, here we examined the effects of orally administered *B. uniformis* CECT 7771 on innate and adaptive immune components of the intestinal epithelium and lamina propria to identify the immune players responsible for its ability to re-establish the metabolic balance in obesity.

## Material and methods

### Culture conditions of *Bacteroides uniformis* CECT 7771

*Bacteroides uniformis* CECT 7771, hereinafter referred to as *B. uniformis*, was identified and grown as previously detailed [21]. To quantify the dose of the bacterium given to mice, vials of the bacterial cells suspensions were plate in Schaedler agar and incubated at 37°C under anaerobic conditions for 48 h to count the colony-forming units (cfu). Some quantified vials were used to obtain dead *B. uniformis* cells through 7 days of exposure of live *B. uniformis* to normal atmospheric oxygen concentrations. The dead of the bacteria was confirmed by plate counting as described above. This method was chosen to maintain the antigenic and immune properties of the bacterial strain. Loss of the ability to grow was confirmed by cultivation in Schaedler agar medium after 48–72-h incubation at 37°C under anaerobic conditions and observing the absence of cfu.

### Mouse studies and metabolic phenotype characterization

Three cohorts of 6-week-old male mice were used to performed the three experiments shown in Figure S1. A total of 100 mice were employed, of which, 30, were used in experiments 1 and 3; and 40, in experiment 2. Sample size (*n* = 10 mice per group) was calculated using the G*Power software based on a previously estimated effect size. Mice, with similar body weight, were randomly allocated into the experimental groups by the animal health care technicians and housed in collective cages (five per cage), with the exception of studies on individual food intake measures, with ad libitum access to water and food, unless otherwise stated. Animals were maintained under constant conditions of humidity and temperature (23 ± 2°C) and regular 12 h of light–dark cycle. Mice were fed a control diet (CD, D12450K containing 10% of energy from fat and without sucrose) or a high-fat high-sugar diet (HFHSD, D12451; 45% of energy from fat and 21% from sucrose) for 14 weeks (both from Research Diets).

### Experiment 1

C57BL/6J wild-type (wt) mice (Charles River Laboratories) fed HFHSD received a daily dose of live *B. uniformis* (5 × 10^8^ cfu per mouse) or vehicle (PBS-0.05% cysteine, 10% glycerol) by oral gavage, whereas CD-fed wt mice received only vehicle. Body weight was monitored weekly and changes in response to nutritional challenge were determined after 6 weeks of HFHSD feeding by measuring body weight loss after a 12-h fast during the dark phase and weight gain and food intake during ad libitum refeeding in the light phase. At 8 weeks of HFHSD feeding, glucose-induced food intake suppression was measured by quantifying the caloric intake during 2 h of refeeding in overnight-fasted mice, after oral administration of saline (0.9%) or glucose (2 g/kg). After 11 and 12 weeks of HFHSD feeding, 4-h fasted mice received an oral load of glucose (2 g/kg) for an oral glucose tolerance test (OGTT), as previously described [21], and to measure glucose-induced secretion of hormones involved in glucose homeostasis and food intake (insulin, GIP, GLP-1, and PYY). For the OGTT, blood was collected from the saphenous vein and glycemia was determined using glucose test strips and a glucometer (Contour® Next meter; Bayer). For the hormone measurements in plasma, blood was obtained from the submandibular vein, collected in Microvette® 500 K3E tubes (Sarstedt) containing inhibitor of dipeptidyl peptidase IV, and immediately centrifuged. After 13 weeks of HFHSD feeding, individual food intake was determined in the light and dark phase (Figure S1A).

### Experiment 2

C57BL/6J mice deficient for *Rag1* and wild-type mice (B6.129S7-Rag1tm1Mom/J #002216) (wt, and *Rag1*^-/-^, respectively) were provided by The Jackson Laboratory (Bar Harbor, ME). Half of the *Rag1*^*-/-*^ and wt mice fed either CD or HFHSD received a daily oral gavage of live *B. uniformis* (5 × 10^8^ cfu per mouse) and half received vehicle. Body weight was monitored weekly and after 11 weeks of HFHSD feeding an OGTT was performed and fasting glycemia was measured. Fecal samples were collected at the end of the experiment and immediately stored at −80°C for later analysis (Figure S1B).

### Experiment 3

C57BL/6J wt mice (Charles River Laboratories) fed HFHSD received a daily dose of dead *B. uniformis* (5 × 10^8^ cfu per mice) or vehicle by oral gavage, while CD-fed wt mice received only vehicle. After 11 weeks of HFHSD feeding, fasting glucose and an OGTT were measured. Body weight was determined at the end of the experiment (Figure S1C).

At the end of each experiment, mice were anesthetized with isoflurane and sacrificed by cervical dislocation. For experiments 1 and 2, anesthetized mice were euthanized by cardiac puncture and blood was collected in Microvette® 500 K3E tubes and immediately centrifuged to obtain plasma. Whole small intestines, except for the first and the last 2 cm, were immediately placed in cold FACS buffer for further isolation of immune cells from the epithelium and the lamina propria. The remaining upper and distal small intestine and the plasma samples were stored at −80°C for later analysis.

All experimental procedures were conducted in the animal facility of the University of Valencia (Spain) and performed in accordance with European Union 2010/63/UE and Spanish RD53/2013 guidelines and were approved by the ethics committee of the University of Valencia (Animal Production Section, SCSIE, University of Valencia, Spain) and authorized by Dirección General de Agricultura, Ganadería y Pesca (Generalitat Valenciana) (approval ID 2018/VSC/PEA/0090 and 2018/VSC/PEA/0171).

### Gut hormone measurements in plasma

Insulin, GIP, and PYY were measured in plasma using multiplex assays on a Luminex® MAGPIX System (Milliplex, Merck Group), and a Multi Species GLP-1 Total ELISA was used to determine GLP-1 in plasma (Merck) in a CLARIOstar Microplate reader (BMG Labtech).

### RNA isolation and RT-qPCR

TRIsure^TM^ reagent (Bioline) was used for total RNA isolation from the duodenum and ileum. Reverse transcription was conducted using 1–2 μg of RNA incubated with MultiScribe^TM^ Reverse Transcriptase (Thermo Fisher Scientific). Appropriate dilutions of cDNA were employed for qPCR using the LightCycler 480 SYBR Green I Master Mix (Roche) and 300 nM of each gene-specific primer pair (Supplementary Table S1) in a LightCycler® 480 Instrument (Roche). Variations in gene expression were calculated based on the 2^−(ΔΔCt)^ method and represented as fold-change expression relative to the control group. Reverse transcription and qPCR conditions are detailed in [21].

### DNA extraction and amplicon-based library construction

The DNA from mice feces was extracted using the QIAamp® PowerFecal® DNA Kit (Qiagen). Bead beating was performed in a Mini-Bead Beater apparatus (BioSpec Products Inc.). The DNA concentration was measured by UV (Nanodrop, Thermo Fisher Scientific). The V3–V4 hypervariable regions of the 16S ribosomal ribonucleic acid (rRNA) gene were amplified using ~10 ng DNA and 25 PCR cycles consisting of the following steps: 95°C for 20 s, 55°C for 20 s, and 72°C for 20 s. Phusion High-Fidelity Taq Polymerase (Thermo Fisher Scientific) and the 6-mer barcoded primers, S-D-Bact-0341-b-S-17 (TAGCCTACGGGNGGCWGCAG) and S-D-Bact-0785-a-A-21 (ACTGACTACHVGGGTATCTAATCC) targeting bacterial 16S rRNA genes [22] were used for PCR. Dual-barcoded PCR products were purified from triplicate reactions with the Illustra GFX PCR DNA and Gel Band Purification Kit (GE Healthcare, Little Chalfont, UK) and quantified using the Qubit 3.0 and the Qubit dsDNA HS Assay Kit (Thermo Fisher Scientific). Samples were multiplexed by combining equimolar quantities of V3–V4 amplicons (~50 ng per sample) and sequenced in one lane of the Illumina MiSeq platform with 2×300 PE configuration (Eurofins Genomics GmbH).

### Microbiota data analysis

Raw data were configured in fastq files and pair ends with quality filtering were assembled using Flash software [23]. Assessment of alpha and beta diversity was performed using an Operational Taxonomic Unit (OTU)-picking approach as follows: sample de-multiplexing was completed with sequence information from barcoded forward/reverse primers and the SeqKit tool [24]. After de-multiplexing and barcodes/primers removal, chimera detection was performed with the UCHIME algorithm [25] and the SILVA reference set of 16S rRNA sequences (Release 138) [26]. The OTU information was retrieved using a rarefied subset of 10,000 sequences per sample, randomly selected after multiple shuffling (10,000×) of the original dataset, and the UCLUST algorithm implemented in USEARCH v8.0.1623 [25]. The alpha diversity descriptors, such as Chao’s index, Simpson’s reciprocal index and phylogenetic distance (PD) were computed using QIIME v1.9.1 [27]. For phylogenetic-based metrics, the OTU sequences were aligned with the PyNAST algorithm and the SILVA aligned reference database. Tree topology reconstruction was retrieved with the FastTree algorithm [28] using the generalized time-reversible (GTR) model and gamma-based likelihood. The community structure across the sample groups was performed with the Vegan R package through interpretative multivariate and constrained appraisal based on the distance-based redundancy analysis (dbRDA) (*vegan::dbrda* function and “bray” method).

For taxonomy assessment, we used the denoising and clustering methods implemented in DADA2 [29] and the complete set of sequences obtained after pair-end assembly with no rarefaction or subsampling. Transformation of compositional amplicon sequence variant (ASV) data was achieved by applying the centered log-ratio (clr) algorithm implemented in *CoDaSeq::codaSeq.clr* with prior execution of *zCompositions:: cmultRepl* function supporting a Bayesian-Multiplicative replacement of count zeros. Taxonomy identification of ASVs was assisted by utilization of DADA2-formatted SILVA database (Release 138).

### In vitro analysis of the immunomodulatory properties of *Bacteroides uniformis* CECT 7771

To develop a co-culture model of human colonic Caco-2 cells and human peripheral blood mononuclear cells (PBMCs), Caco-2 cells were first cultured in Eagle’s minimum essential medium (ATCC) with 10% heat-inactivated fetal bovine serum (FBS; Gibco/ Thermo Fisher Scientific), penicillin (100 U/mL), streptomycin (100 μg/mL), and amphotericin B (0.25 μg/mL) (all from Sigma‐Aldrich, Madrid, Spain). In total, 1 × 10^5^ cells (passage 12) per well were seeded in a 12-well plate containing 12 mm polycarbonate inserts with a pore size of 0.4 μm (Corning™ Transwell™, Thermo Fisher Scientific). When the trans-epithelial electrical resistance, measured with a Millicell-ERS-2 voltohmmeter (Merck), reached 500–600 Ω cm^2^, 1 × 10^6^ PBMC (Stem Cell Technologies) were seeded in the basolateral compartment in the same supplemented medium. Caco-2 cells were exposed to *B. uniformis* (1/10 ratio of Caco-2/ bacterial cells) or PBS 24-h later for 16 h.

To generate primary cultures of naïve CD4+ T cells, the spleen and lymph nodes from the inguinal adipose tissue of 6-week-old male C57BL/6J mice were harvested and immediately placed in PBS-10% FBS. Tissues were homogenized, filtered (70-µm nylon cell strainers, Biologix), and centrifuged (450 g, 5 min, 4°C). Erythrocytes were removed from spleen by incubating cells with lysis buffer (150 mM NH_4_Cl, 10 mM KHCO_3_, and 0.1 mM EDTA). Cells from spleen and lymph nodes were combined to isolate naïve CD4+ T cells using a naïve CD4+T cell isolation kit and LS MACS columns (Miltenyi Biotec). Cells were seeded in anti-CD3 (2.5 μg/mL) pre-coated 96-well plate (3 × 10^5^ cells per well) and cultured for 4 days in TexMACS medium (Miltenyi Biotec) with 5% heat-inactivated FBS, penicillin (100 U/mL), streptomycin (100 μg/mL), TGFβ (2 ng/mL), IL2 (50 U/mL), and anti-CD28 (0.5 μg/mL) for antigen-induced activation, T cell proliferation, and Treg differentiation [30]. Cells were then incubated with *B. uniformis* (1/10 ratio of naïve CD4+ T cell/bacterial cells) or PBS for 16 h.

### Flow cytometry

Cleaned small intestines were longitudinally opened and cut into small pieces. To isolate intestinal epithelial cells, the tissue was incubated twice in Hank’s balanced salt solution (HBSS) with calcium and magnesium (Thermo Fisher Scientific) supplemented with 5 mM EDTA (Scharlab), 1 mM DTT, 100 µg/mL streptomycin, and 100 U/mL penicillin (Merck, Germany) in an orbital shaker for 30 min at 37°C. The obtained supernatant fractions were filtrated using 100-µm nylon cell strainers (Biologix) and centrifuged. The isolated epithelial cells were preserved in FACS buffer (PBS with 0.5% BSA) until immunolabeling. To isolate lamina propria cells, the remaining tissue was washed with PBS and incubated twice in HBSS supplemented with 0.5 mg/mL collagenase D (Roche Diagnostics GmbH, Basel, Switzerland), 3 mg/mL dispase II (Sigma‐Aldrich), 1 mg/mL DNase I (Roche), 100 µg/mL streptomycin, and 100 U/mL penicillin under orbital agitation at 37°C for 30 min. To harvest cell suspensions, the supernatant fractions were filtered through 70-µm nylon cell strainers, centrifuged, and preserved in FACS buffer. Isolated cells were incubated with different immune markers for 30 min at 4°C in darkness to measure natural and induced intraepithelial lymphocytes (IELs) in the epithelium and pro- and anti-inflammatory macrophages (M1 and M2, respectively) and T cells from the lamina propria. Immunolabeling was conducted using the antibodies for flow cytometry detailed in Supplementary Table S2. For intracellular marker staining, cells were first permeabilized and fixed (fixation/permeabilization solution kit, BD Bioscience).

Flow cytometry was also employed to analyze the in vitro immunomodulatory properties of *B. uniformis*. In brief, after the 16-h *B. uniformis* exposure, PBMCs from the human co-culture model with Caco-2 cells, and CD4+ T primary cells, were collected to quantify CD4+ T cells and regulatory T cell (Tregs). PBMCs were immunolabeled with BV510 anti-CD4, PE-conjugated anti-CD25 and Alexa Fluor® 647 anti-FOXP3 anti-human antibodies (all from BD Biosciences), whereas VioBlue-conjugated anti-CD4 and PE-conjugated anti-Foxp3 anti-mouse antibodies were used to immunolabel CD4+ T primary cultured cells (all from Miltenyi Biotec). Before antibody incubation, cells were incubated with the FcR Blocking Reagent (Miltenyi Biotec). In addition, cells were permeabilized and fixed (fixation/permeabilization solution kit; BD Bioscience) to allow intracellular staining (FOXP3).

Data acquisition and analysis were performed using a BD LSRFortessa flow cytometer operated with FACS Diva software v.7.0 (BD Biosciences).

### Statistical analysis

GraphPad Prism 9 (GraphPad Software Inc.) was used for statistical analysis and for graph plotting, with the exception of gut microbiota-related data. Normal distributed data with equal variances, identified by the Shapiro–Wilk test and Bartlett's test, respectively, were analyzed using different parametric tests. For the experiments including three groups (experiment 1 and 3), statistics were performed using paired *t*-tests, one-way ANOVA, and two-way ANOVA with experimental groups as the between-subject factor and time as the within-subject factor. Student’s *t*-test was employed for analyses restricted to HFHSD-fed mice. In experiment 2, two-way ANOVA was conducted with genotype (wt or *Rag1*^*-/-*^) and diet (CD or HFHSD) as the between-subject factors (statistics shown in Figure S4) and genotype and treatment (vehicle or *B. uniformis*) as the between-subject factors (statistics shown in Figs. 3 and 4).

Post hoc analyses were conducted using Tukey or Bonferroni corrections only when interactions were identified in the two-way ANOVA. The Kruskal–Wallis test followed by pairwise multiple comparisons was used to analyze non-normally distributed data. The Mann–Whitney *U* test was used for analysis restricted to HFHSD-fed mice. Bravais–Pearson’s correlation coefficient was used to test correlations between two variables.

Alpha diversity descriptors of the gut microbiota-related data were analyzed by non-parametric methods such as Kruskal–Wallis and pairwise Wilcoxon rank sum tests (for unpaired samples) with the Benjamini–Hochberg post hoc correction for multiple group comparisons. Differences in the microbial community structure were assessed using the permutation-based *vegan::adonis* function. Differential abundance analysis of ASVs across groups was assisted by applying the Kruskal–Wallis test with the Benjamini–Hochberg post hoc correction. Highly divergent ASVs in terms of abundance were selected with Kruskal–Wallis corrected *p*-value ≤ 0.01. Graphs and plots of the gut microbiota-related data were drawn using the ggplot2 package in R v3.6.

All animals were included in the analyses except in the case of outcome measures out of the detection ranges of the aforementioned techniques.

## Results

### *Bacteroides uniformis* prevents obesity and improves glucose sensing in obese mice

**Fig. 1:**
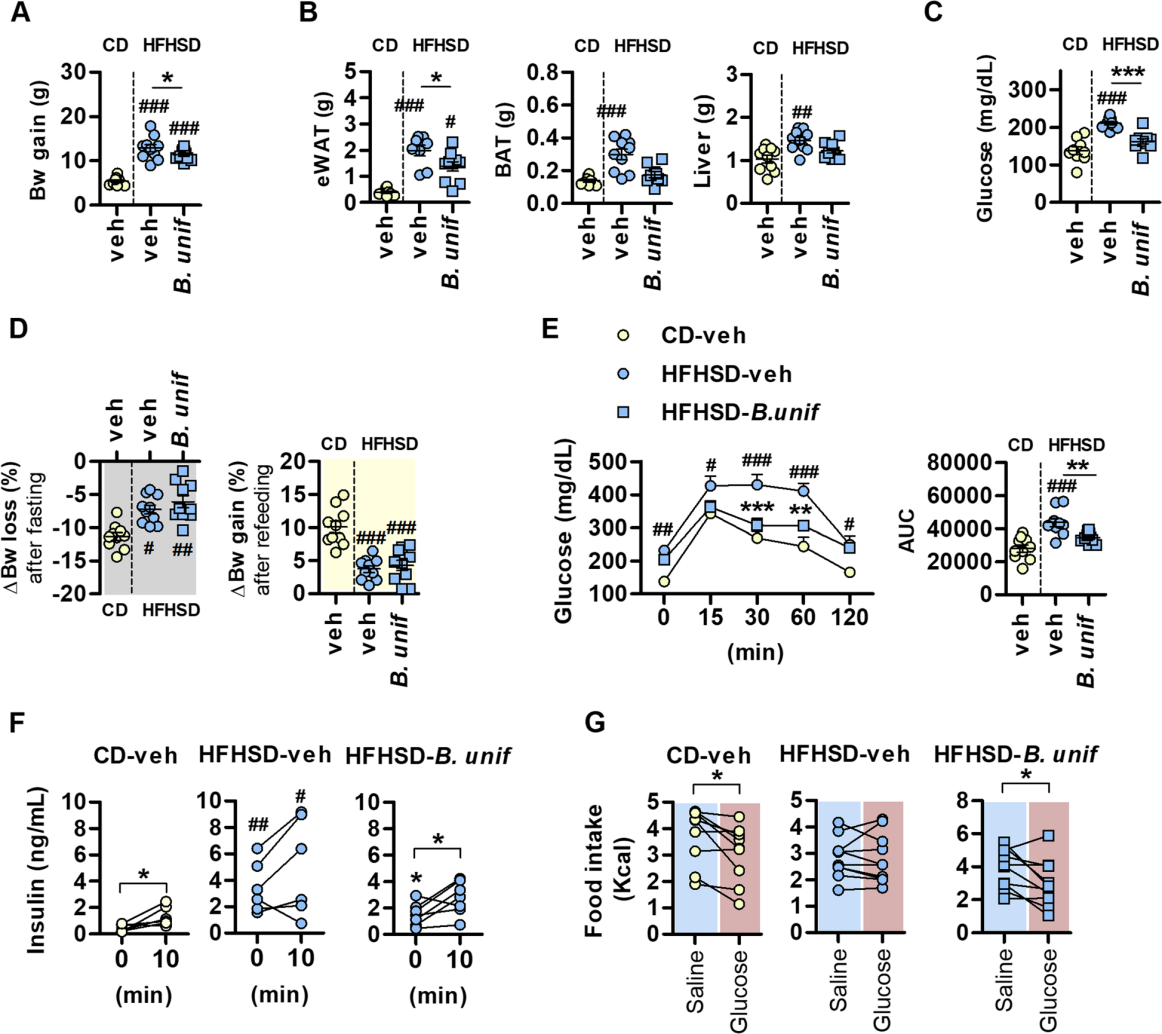
Impact of *Bacteroides uniformis* on the metabolic phenotype of diet-induced obese mice. **A** Body weight (Bw) gain, **B** weight of epididymal white and brown adipose tissue (eWAT and BAT, respectively) and liver, and **C** fasting glucose levels, in mice fed a control diet (CD) or a high-fat high-sugar diet (HFHSD) plus vehicle (veh), or HFHSD plus a daily oral dose of *B. uniformis* (*B.unif*) for 14 weeks; **D** percentage of Bw variation (Bw loss and gain) after overnight fasting and 12-h refeeding during the light phase after 6 weeks of HFHSD/CD-feeding; **E** glycemia at 15, 30, 60, and 120 min in response to an oral glucose load in mice fed HFHSD or CD for 14 weeks; **F** oral glucose-induced insulin secretion and **G** oral glucose-induced caloric intake suppression after 12 or 8 weeks of HFHSD/CD-feeding, respectively. Results are represented by scatter plots indicating individual values or summary data with mean ± SEM, (*n* = 6–10 per group). One-way ANOVA followed by Tukey’s post hoc test: **A**, **C**, **D**, **F**, and **G**, eWAT and liver in **B** and AUC in **E**. Kruskal–Wallis test followed by Dunn’s multiple comparison test: BAT in **B**. Two-way ANOVA with group as between-subject factor and time as within-subject factor followed by Bonferroni’s post hoc test comparing replicate means by time: **E**. Paired *t* tests: **F** and **G** with time as within-subject factor. *p < 0.05, **p < 0.01, and ***p < 0.001 for comparisons indicated by the horizontal line. ^#^*p* < 0.05, ^##^*p* < 0.01, and ^###^*p* < 0.001 for HFHSD-fed groups vs CD-veh

Oral administration of *B. uniformis* suppressed the body weight gain of mice fed HFHSD (Fig. 1A and S2A) and prevented (totally or partially) the increase in epididymal white adipose tissue (eWAT), brown adipose tissue (BAT), and liver mass (Fig. 1B), but not inguinal WAT (iWAT) (Figure S2B) (scheme of the intervention in Figure S1A). Likewise, the fasting hyperglycemia induced by HFHSD was improved in mice administered *B. uniformis* (Fig. 1C). The suppression of weight gain by *B. uniformis* administration was not associated with functional improvements in body weight control in response to a nutritional challenge evaluated in a fasting-refeeding test. Compared with mice on CD, both untreated and *B. uniformis*-treated HFHSD-fed mice showed resistance to lose weight in response to overnight fasting and reduced weight gain in the refeeding period (Fig. 1D). *B. uniformis*-treated HFHSD-fed mice showed improved oral glucose tolerance (Fig. 1E) along with reduction of the HFHSD-induced hyperinsulinemia and restoration of insulin secretion after an oral glucose challenge (Fig. 1F). We ruled out the possibility that the benefits of *B. uniformis* on glucose homeostasis were the result of improvements in incretin hormones, as the levels of GLP-1 and GIP in fasting conditions and after oral glucose challenge did not differ between treatments (Figure S2C, S2D).

The ability of *B. uniformis* to prevent body weight gain was independent of ad libitum caloric intake, which was unchanged between untreated and *B. uniformis*-treated mice (Figure S2E). Nonetheless, untreated mice on HFHSD showed an altered food intake after overnight fasting, with a higher caloric intake than CD-fed mice after 6 h of ad libitum refeeding (Figure S2F). *B. uniformis* administration attenuated the enhanced fasting-induced feeding under HFHSD, as the caloric intake of mice receiving the bacterium was similar to that of CD-fed mice (Figure S2F). Focusing on glucose-related effects on feeding behaviour, we found that an oral glucose load failed to reduce food intake in untreated HFHSD-fed mice, but effectively suppressed feeding in *B. uniformis*-treated mice (Fig. 1G), which suggests improved intestinal glucose sensing. Peptide YY (PYY) was not involved in the ameliorating effect of *B. uniformis* on glucose-induced food intake suppression, as its levels remained unchanged after fasting and after the oral glucose load whatever the group (Figure S2G).

### *Bacteroides uniformis* administration suppresses obesity-induced inflammation in the small intestine and metabolic endotoxemia

**Fig. 2:**
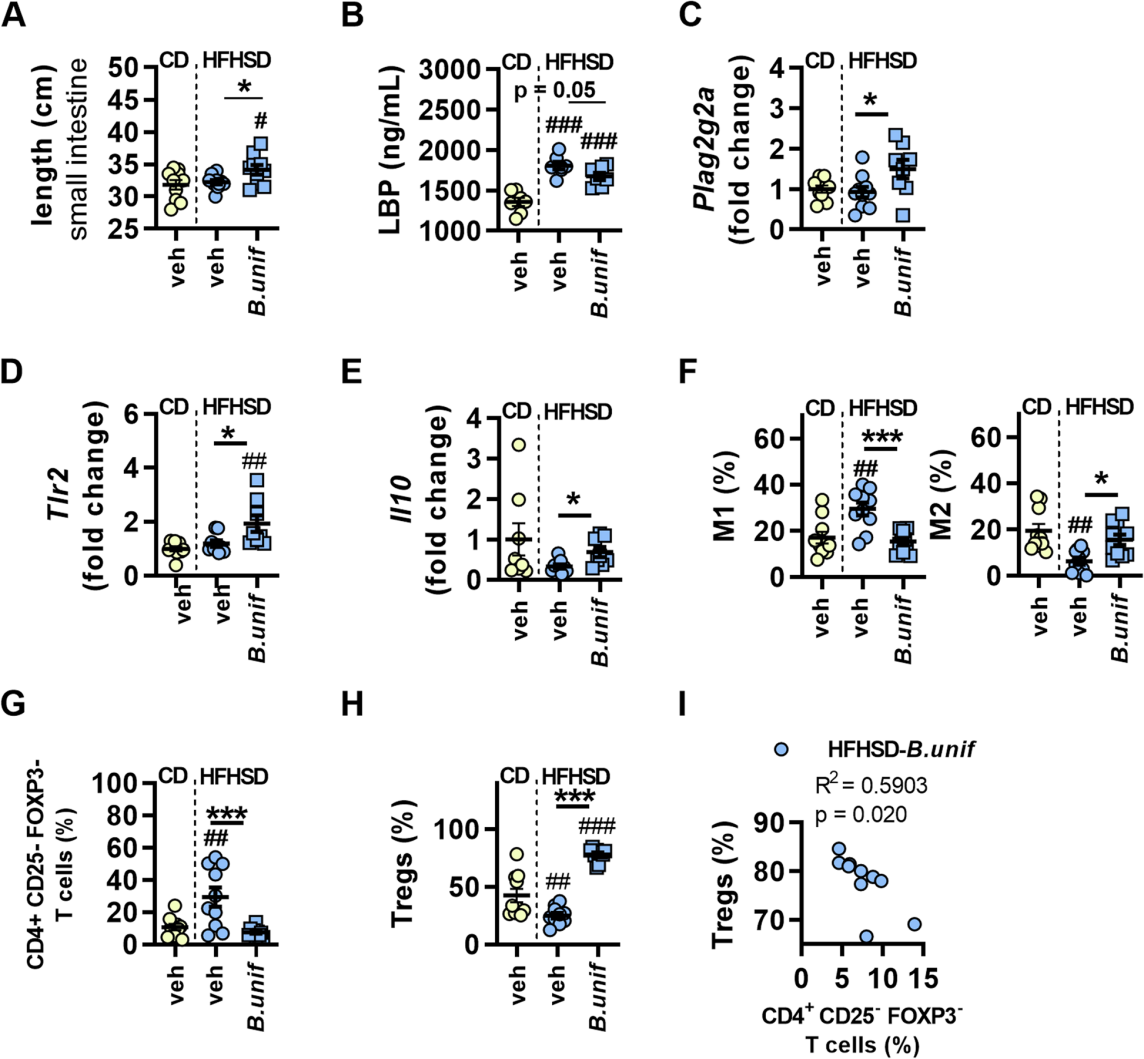
Effects of *Bacteroides uniformis* on metabolic endotoxemia and intestinal immunity in diet-induced obese mice. **A** Small intestine length, **B** LPS-binding protein (LBP) levels in plasma; gene expression of **C** *Plag2g2a*, **D** *Tlr2*, and **E** *Il10* in ileum; percentage of **F** type 1 and type 2 macrophages, **G** CD4^+^ T (CD25^-^ FOXP3^-^) cells, and **H** regulatory T cells (Tregs) in the lamina propria of the small intestine, and **I** correlation between CD4^+^ T (CD25^-^ FOXP3^-^) and Tregs in mice fed a control diet (CD) or a high-fat high-sugar diet (HFHSD) plus vehicle (veh) or HFHSD plus a daily oral dose of *B. uniformis* (*B.unif*) for 14 weeks. Results are represented by scatter plots with mean ± SEM, (*n* = 7–10 per group). One-way ANOVA followed by Tukey’s *post hoc* test: **A**–**C**, M1 in **F**, **G**, and **H**; Student´s *t*-test for HFHSD-fed groups: **B** and **E**; Kruskal–Wallis test followed by Dunn's multiple comparison test: **D** and M2 in **F**; Mann–Whitney *U* test for HFHSD-fed groups: **D**; and Pearson correlation: **I**. *p < 0.05 and ***p < 0.001 for comparisons indicated by the horizontal line. ^#^*p* < 0.05, ^##^*p* < 0.01, and ^###^*p* < 0.001 for HFHSD-fed groups vs CD-veh

We explored the potential metabolic and immune routes stemming from the gut that could be responsible for the *B. uniformis-*induced metabolic improvements in obesity. The small intestine was longer in obese mice receiving *B. uniformis* than in CD-fed mice (Fig. 2A), and analysis of HFHSD-fed groups revealed that administration of the bacterium increased the length of the small intestine (Fig. 2A). By contrast, the duodenal transcript levels of the glucose transporters *Slc2a* and *Slc5a1* in HFHSD-fed mice receiving or not *B. uniformis* were similar to those of CD-fed mice (Figure S3A).

HFHSD feeding increased the plasma levels of LPS-binding protein (LBP), and administration of *B. uniformis* slightly attenuated the metabolic endotoxemia (Fig. 2B). Compared with untreated HFHSD-fed mice, *B. uniformis* administration induced an increase in the ileal gene expression of the secretory group IIA phospholipase A2 (*Plag2g2a*), which has bactericidal activity (Fig. 2C and S3B). Expression analysis of the pathogen sensors toll-like receptors (TLRs) and nucleotide-binding oligomerization domain (NOD) proteins, and cytokines, revealed that *B. uniformis* administration enhanced the ileal expression of *Tlr2* and the anti-inflammatory cytokine* Il10* (Fig. 2D, E and S3C). *B. uniformis* administration had no effect on the abundance of natural or induced IELs in the epithelium (Figure S3D), but influenced macrophages and CD4^+^ T cells in the lamina propria (Fig. 2F–I). Notably, *B. uniformis* administration normalized the altered levels of M1 and M2 induced by HFHSD feeding (Fig. 2F). Likewise, its administration reversed the increased abundance of CD4^+^ CD25^-^ FOXP3^-^ T cells (Fig. 2G) and the reduced levels of Tregs (Fig. 2H) induced by HFHSD feeding. Indeed, a positive correlation was identified between CD4^+^ CD25^-^ FOXP3^-^ T cells and Tregs exclusively in mice receiving *B. uniformis* (Fig. 2I and Figure S3E).

### *Bacteroides uniformis* requires adaptive immunity to improve oral glucose tolerance

Our results thus far show that *B. uniformis* enhances the abundance of Tregs and M2 in the lamina propria of the small intestine in obese mice. It is known that while M2 contribute to limit antigen-induced pro-inflammatory responses in the gut [31], adaptive immune cells more efficiently counteract the response mediated by pro-inflammatory signals [32]. To understand the extent to which the reduced intestinal inflammatory tone and the associated metabolic benefits of *B. uniformis* administration in obesity depend on either M2 or Tregs induction, we next performed studies in a mouse model of deficient adaptive immunity (*Rag1*^*-/-*^ mice) (scheme of the intervention in Figure S1B).

Adaptive immunity did not influence the development of the obese phenotype, as body weight gain, eWAT, iWAT, and BAT and liver weight, small intestine length, and oral glucose tolerance were similarly affected by HFHSD in wt and *Rag1*^*-/-*^ mice receiving vehicle (Figure S4A–S4G).

**Fig. 3:**
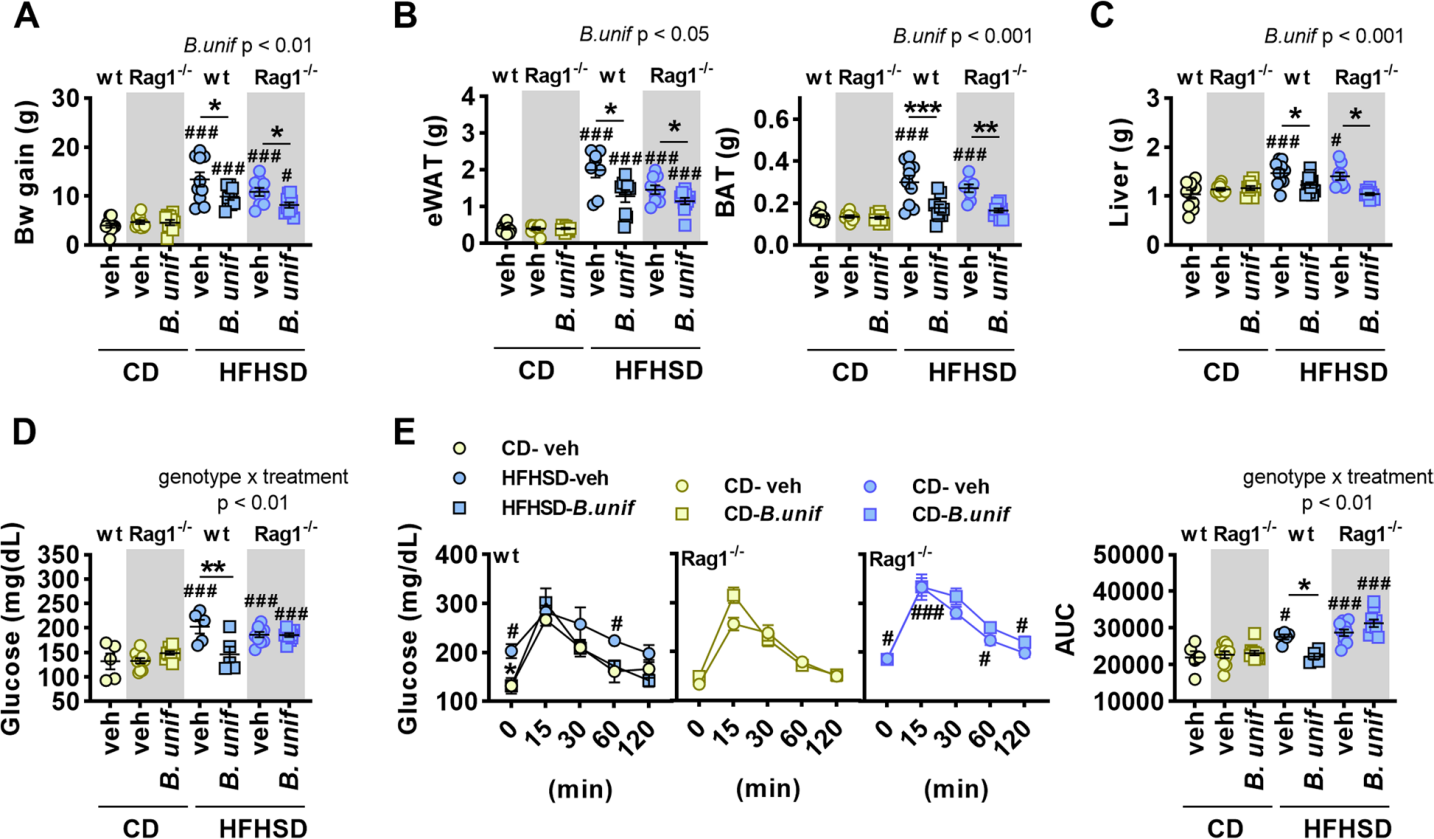
Effects of *Bacteroides uniformis* on the metabolic phenotype of *Rag1*-deficient mice. **A** Body weight (Bw) gain; weight of **B** epidydimal (eWAT) and brown adipose tissue (BAT) and **C** liver; **D** fasting glucose levels in plasma and **E** oral glucose tolerance showing glycemia throughout the time in response to an oral glucose load and the area under the curve (AUC) in wild type (wt) and *Rag1*^*-/-*^ mice fed either control diet (CD) or high-fat high-sugar diet (HFHSD) orally receiving vehicle (veh) or *B. uniformis* (*B.unif*). Results are represented by scatter plots indicating individual values or summary data with mean ± SEM (*n* = 5–10 per group). One-way ANOVA followed by post hoc Tukey´s test vs CD-veh mice in either *Rag1*^*-/-*^ or wt mice. Two-way ANOVA in HFHSD-fed groups with genotype (wt or *Rag1*^*-/-*^) and treatment (vehicle or *B. uniformis*) as between-subject factors followed by Tukey’s post hoc test when interactions were identified. Student’s *t* test in either *Rag1*^*-/-*^ or wt mice was conducted when main effects were identified. *p < 0.05 and **p < 0.01 for comparisons indicated by the horizontal line. ^#^*p* < 0.05 and ^###^*p* < 0.001 for HFHSD-fed groups vs CD-veh in either *Rag1*^-/-^ or wt mice

The analysis restricted to HFHSD groups revealed a main effect of *B. uniformis* on body weight gain and the weight of eWAT, BAT, and liver (Fig. 3A–C), without affecting inguinal fat (data not shown). In particular, similar to wt mice*, B. uniformis* administration to *Rag1*^*-/-*^ mice on HFHSD limited the weight gain (Fig. 3A) and the fat accumulation in the eWAT, BAT and liver (Fig. 3B, C). An interaction between the genotype and the treatment was identified on fasting glucose in plasma (Fig. 3D) and area under the curve (AUC) of the OGTT (Fig. 3E). This interaction indicated that, while in wt mice *B. uniformis* reduced both, fasting glycemia and the AUC, it failed to alleviate hyperglycemia and oral glucose intolerance in *Rag1*^*-/-*^ mice (Fig. 3D and E), overall suggesting that adaptive immunity is required for the *B. uniformis*-mediated effects on glucose homeostasis but not for limiting fat depots. The administration of *B. uniformis* normalized hyperinsulinemia in *Rag1*^*-/-*^ mice fed HFHSD (Figure S5).

### *Bacteroides uniformis* fails to induce M2-mediated anti-inflammatory responses in the intestine of obese Rag1-/- mice

**Fig. 4:**
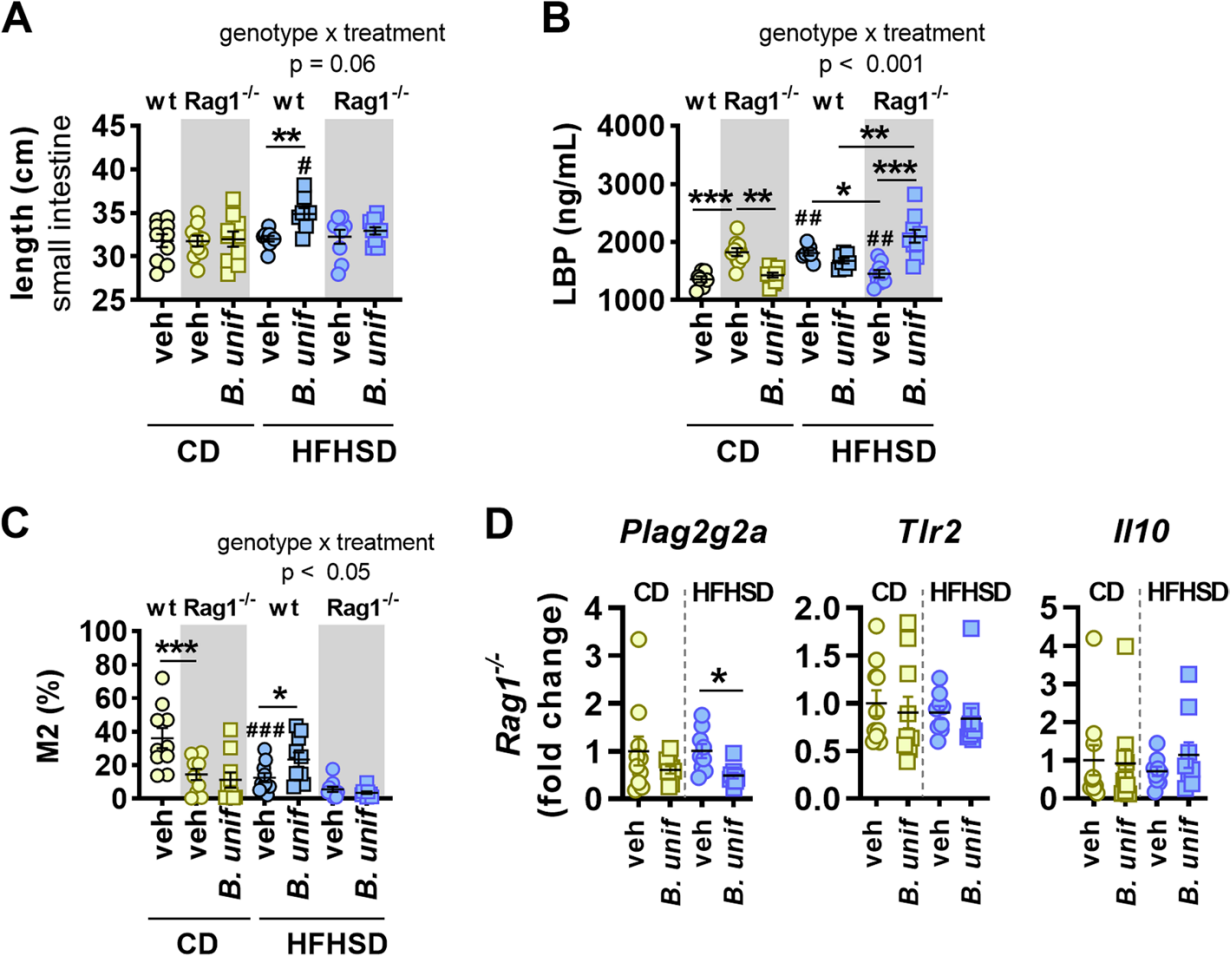
*Bacteroides uniformis* effects on the small intestinal mucosa of *Rag1*-deficient mice. **A** Small intestine length; **B** lipopolysaccharide-binding protein (LBP) levels in plasma; and **C** percentage of type 2 macrophages (M2) in the lamina propria of the small intestine of wild type (wt) and *Rag1*^*-/-*^ mice fed either control diet (CD) or high-fat high-sugar diet (HFHSD) orally receiving vehicle (veh) or *B. uniformis* (*B.unif*). **D** mRNA levels of *Plag2g2a, Tlr2*, and *Il10* in ileum of *Rag1*^*-/-*^ mice fed either CD or HFHSD orally receiving veh or *B.unif*. Results are represented by scatter plots indicating individual values with mean ± SEM, (*n* = 7–10 per group). One-way ANOVA followed by post hoc Tukey’s test vs CD-veh mice in either *Rag1*^*-/-*^ or wt mice. Two-way ANOVA in HFHSD-fed groups with genotype (wt or *Rag1*^*-/-*^) and treatment (vehicle or *B. uniformis*) as between-subject factors followed by Tukey’s post hoc test when interactions were identified. *p < 0.05 and **p < 0.01 for comparisons indicated by the horizontal line. ^#^*p* < 0.05 and ^###^*p* < 0.001 for HFHSD-fed groups vs CD-veh in either *Rag1*^-/-^ or wt mice

The absence of adaptive immunity inhibited the *B. uniformis-*induced increase in the length of the small intestine of HFHSD-fed mice (Fig. 4A). In CD-fed groups receiving vehicle, the loss of adaptive immunity increased plasma LBP levels (wt vs *Rag1*^*-/-*^) and *B. uniformis* administration blunted this increase (Fig. 4B). Compared with CD-fed groups, HFHSD feeding has contrasting effects depending on genotype, inducing metabolic endotoxemia in wt mice, and attenuating the LBP levels in plasma in *Rag1*^*-/-*^ mice (Fig. 4B). The analysis restricted to HFHSD-fed mice indicated an interaction between the genotype and the treatment. In particular, this interaction also indicated contrasting effects of *B. uniformis* on LBP levels that were genotype dependent: in wt mice, *B. uniformis* tended to reduce the LBP levels (*p* = 0.05), whereas in *Rag1*^*-/-*^, the bacterium increased these levels (Fig. 4B). In addition, compared with wt mice on CD, the absence of adaptive immunity decreased the abundance of M2 in the lamina propria of mice receiving vehicle (Fig. 4C). The analysis restricted to HFHSD-fed mice revealed an interaction between the genotype and the treatment; the post hoc analysis indicated that *B. uniformis* administration failed to increase the levels of M2 in Rag1^-/-^ mice as did in wt mice (Fig. 4D). The exploration of the gene expression of intestinal immune markers only in *Rag1*^*-/-*^ mice revealed that administration of *B. uniformis* to these mice had opposite or not effects compared to those showed in wt mice. In contrast to wt mice on HFHSD (Fig. 2C–E), *B. uniformis* reduced the transcript levels of the bactericidal enzyme gene *Plag2g2a* in *Rag1*^*-/-*^ mice (Fig. 4D) and had no effect on the ileal gene expression of *Tlr2* and *Il10* (Fig. 4D). Altogether, the results indicate that *B. uniformis* requires adaptive immunity to increase the abundance of M2 in the small intestine of diet-induced obese mice. The findings also suggest that adaptive immunity weakens primary gut barrier integrity, differentially affecting the response to commensal bacteria depending on diet, either increasing or reducing endotoxemia.

### *Bacteroides uniformis* has only a minor impact on gut microbiota in obese wild-type mice and slightly exacerbates the reduced diversity in obese Rag1-deficient mice

Changes in gut microbiota could partly underlie the loss of effects of *B. uniformis* in promoting anti-inflammatory responses in the small intestine and improving glucose homeostasis in obese mice lacking adaptive immunity. To test this, we examined the interactions between genotype (wt and *Rag1*^-/-^) and diet (CD and HFHSD) on alpha and beta diversity in mice receiving vehicle. The main variable explaining changes in alpha diversity of the fecal microbiota was the *Rag1* genotype (Figure S6A). Indeed, the absence of this gene decreased the richness, weighted diversity and phylogenetic diversity (PD) in both CD- and HFHSD-fed mice (Figure S6A). The obesogenic diet did not significantly worsen the effect of the *Rag1* gene deletion on diversity (Figure S6A), with only a slightly larger reduction in the PD index in HFHSD- compared with CD-fed *Rag1*^*-/-*^ mice (median HFHSD-PD_Δ_ = −4.95, *p* = 0.089 vs median CD-PD_Δ_ = −2.18, *p* = 0.151) (Figure S6A). Analysis of beta diversity indicated that all possible combinations of diet (CD and HFHSD) and genotype (wt and *Rag1*^*-/-*^) in groups treated with vehicle induced profound changes in fecal microbial community structure, resulting in four different microbiota configurations (Adonis = 14.5, *p* < 0.001) (Figure S6B).

**Fig. 5:**
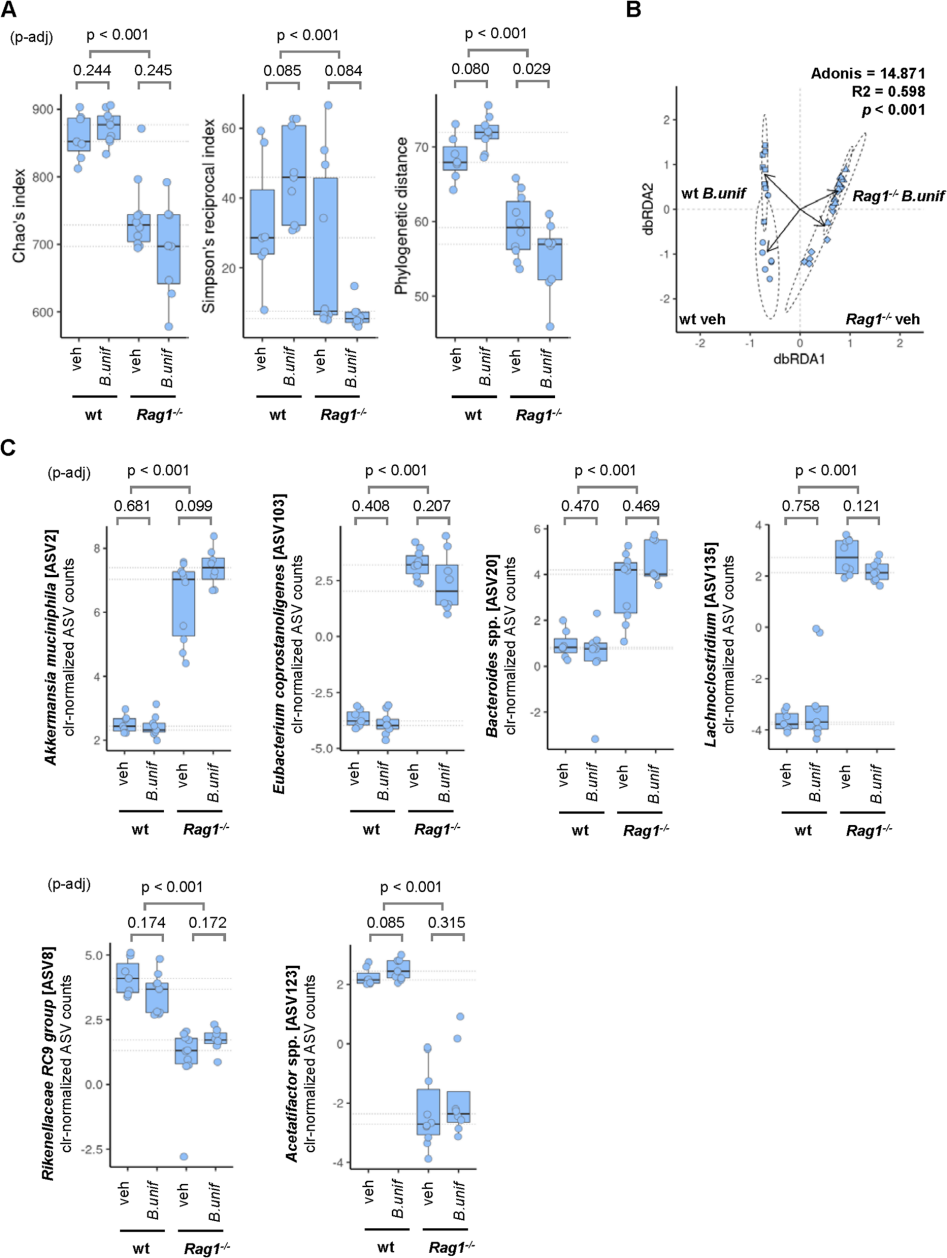
Effects of *B. uniformis* administration on alpha and beta diversity and bacterial taxonomy of the intestinal microbiota. **A** Chao’s richness, Simpson’s reciprocal index, and phylogenetic distance estimators were calculated for wild-type (wt) and *Rag1*-deficient (*Rag1*^*-/-*^*)* mice fed a high-fat high-sugar diet (HFHSD) plus either vehicle (veh) or *B. uniformis* (*B.unif*) daily orally for 14 weeks. Wilcoxon rank sum test after Shapiro–Wilk normality test was applied for comparison between groups. Benjamini–Hochberg post hoc correction was applied to multiple group pairwise comparisons (p-adj) when investigating genotype × treatment interactions. Data distributions are shown in boxplots including the different data points. Medians for all groups are represented by dotted grey lines projected from *y*-axis. **B** Constrained distance-based (Bray–Curtis) redundant analysis (db-RDA) to determine changes in microbiota structure according to genotype (wt *or Rag1*^*-/-*^) or treatment (veh or *B.unif*). Results of Adonis test for groups comparison is shown within the scatter plot as well as the group labels. The two most informative dimensions of dbRDA are shown. **C** Six amplicon sequence variants (ASVs) with larger size-effect and taxonomy identity at genus or species level in the intestine of wt and *Rag1*^*-/-*^ mice receiving vehicle or *B. uniformis*. The pairwise Wilcoxon rank sum test was applied for comparisons among groups. *p*-values on top of respective panels correspond to adjusted *p*-values following post hoc correction (Benjamini–Hochberg). Medians for all groups are represented by dotted grey lines projected from *y*-axis

As the genotype rather than the diet profoundly affected alpha diversity, we further explored the potential differential effects of *B. uniformis* administration dependent on genotype under an obesogenic diet (Fig. 5). We noted a trend for an increase in PD in HFHSD-fed wt mice administered *B. uniformis* (median PD_Δ_ = 4.02, *p* = 0.080), understood as a gain of microbial species, but this parameter was lower in HFHSD-fed *Rag1*^*-/-*^ mice administered *B. uniformis* than in that administered vehicle (median PD_Δ_ = −2.25, *p* = 0.029) (Fig. 5A).

In HFHSD-fed wt mice, the microbiota community structure differed between groups receiving or not *B. uniformis* (Fig. 5B). Nevertheless, *Rag1*^*-/-*^ mice under HFHSD feeding, either treated with vehicle or *B. uniformis,* had a microbiota configuration less divergent than that observed in wt groups (Adonis = 14.8, *p* < 0.001) (Fig. 5B).

A large number of microbial species defined as amplicon sequence variants (ASVs) showed differences in their abundance between the different experimental groups. Of those, we specifically considered the ASVs that could be taxonomically identified (at genus or species level) and that show large-size changes (based on the non-parametric Kruskal–Wallis test) in the different comparisons (between diet and genotype and between genotype and *B. uniformis*). Figure S6C shows the ASVs significantly influenced by the diet (CD or HFHSD) and the genotype (wt or *Rag1*^*-/-*^) while Fig. 5C shows ASVs influenced by *B. uniformis* in wt and *Rag1*^*-/-*^ mice under HFHSD feeding.

In line with the results for diversity, the major driver of the microbiota compositional changes was the genotype rather than the diet (Figure S6C). *Akkermansia muciniphila* (ASV2), *Eubacterium coprostanoligenes* (ASV103), *Bacteroides* spp. (ASV20), and *Lachnoclostridium* (ASV135) were greater in abundance in *Rag1*^*-/-*^ mice, whereas *Rikenellaceae-RC9* group (ASV8) and *Acetatifactor* spp. (ASV123) were lower (Figure S6C). Compared with CD-fed mice, HFHSD feeding induced opposite effects on the beneficial intestinal bacterium *A. muciniphila* (ASV2) in the different genotypes, reducing its abundance in wt mice (clr_Δ_ = −1.43, *p* = 0.012) but further increasing its abundance in *Rag1*^*-/-*^ mice (clr_Δ_ = 2.20, *p* = 0.015) (Figure S6C). Moreover, HFHSD feeding further boosted the abundance of the cholesterol-transforming *E. coprostanoligenes* (ASV103) in mice lacking adaptive immunity but had no effects in wt mice (Figure S6C). *B. uniformis* administration failed to exert any detrimental effects on *A. muciniphila* or *E. coprostanoligenes* abundance in either in wt or *Rag1*^*-/-*^ mice under HFHSD, beyond that caused by the genotype (Fig. 5C). These results indicate that *B. uniformis* does not significantly affect the abundance of specific bacterial species under an obesogenic diet and only amplifies the reduced alpha diversity of adaptive immunity deficient mice.

### *Bacteroides uniformis* directly induces regulatory T cells and requires viability to prevent body weight gain but not to exert glucoregulatory effects on obese mice

The only ASV identified as *B. uniformis* (ASV15) had a greater abundance in *Rag1*^*-/-*^ mice on CD than in all other groups (*p* < 0.001), and neither HFHSD nor *B. uniformis* administration significantly affected its abundance (data not shown). This suggests that the transit of *B. uniformis* through the gastrointestinal tract is sufficient to restore intestinal immune fitness and improve oral glucose tolerance and that engraftment is not indispensable. Based on this evidence and on the limited impact of *B. uniformis* administration on the abundance of other bacteria (Fig. 5C), we hypothesized that *B. uniformis* could directly modulate CD4+ T cells during its transit through the small intestine to help mitigate HFHSD-induced inflammation and glucose intolerance. We addressed this by exploring two possible immunomodulatory paths dependent or not on intestinal epithelial cells: first, that the bacterium stimulates intestinal epithelial cells with downstream effects on CD4+ T cells and, second, that the bacterium directly interacts with naïve CD4+ T cells priming their differentiation into Tregs.

**Fig. 6:**
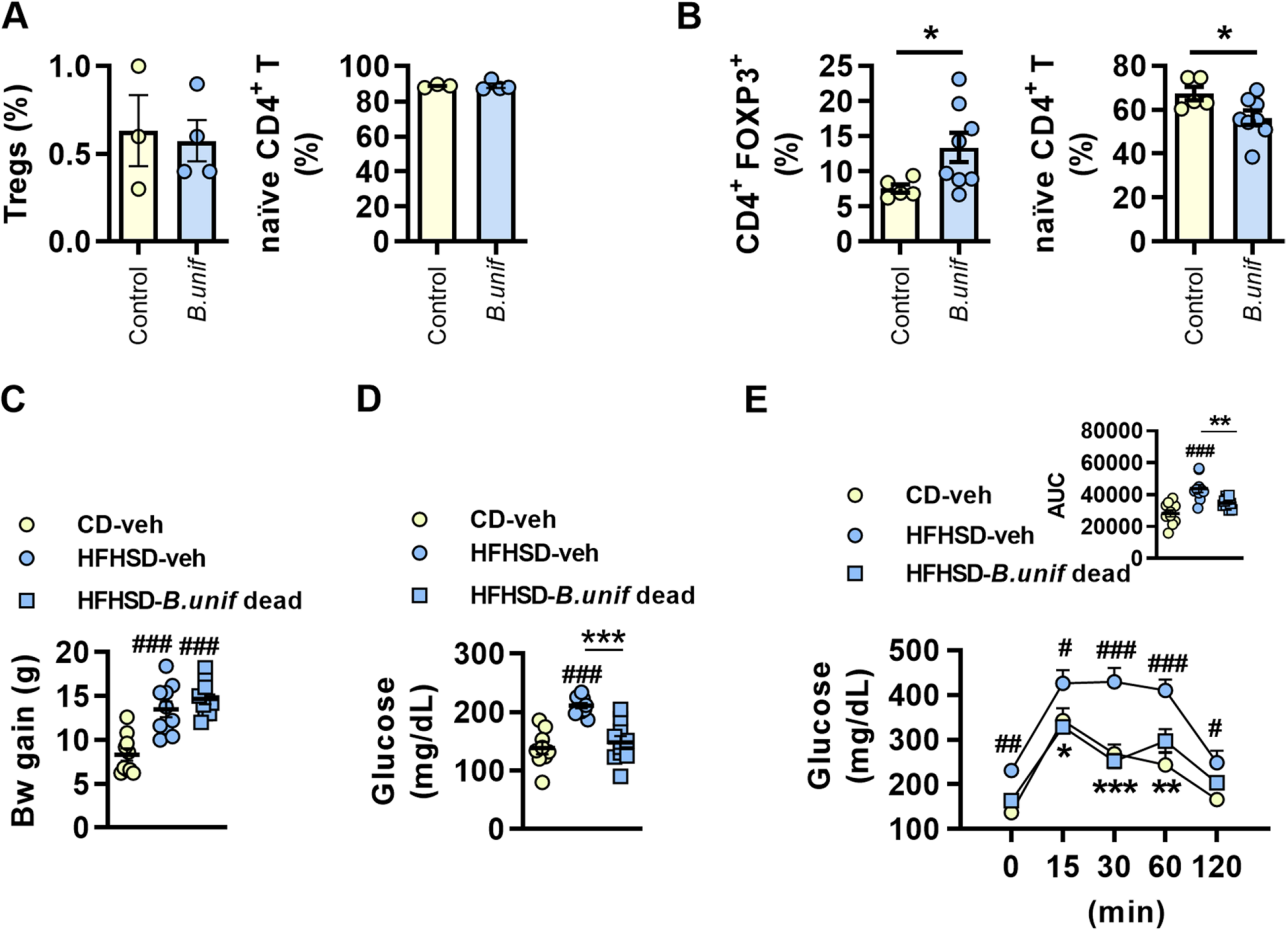
Immunomodulatory properties of *Bacteroides uniformis* in vitro and metabolic effects of the live and dead bacterium in diet-induced obese mice. **A** Percentage of regulatory T cells (Tregs) and naïve CD4+ T cells in human peripheral blood mononuclear cells co-cultured with human intestinal colonic epithelial cell monolayers (Caco-2) stimulated or not with *Bacteroides uniformis* (*B.unif*) (*n* = 3 wells per group). **B** Percentage of Tregs and naïve CD4+ T cells in primary cultures of splenic and lymphoid nodes naïve CD4+ T cells stimulated or not with *B. uniformis* (*n* = 5–8 wells per group). Two different experiments were conducted (*n* = 6 mice). Graphs represent results from one experiment. **A**, **B** Individual values are represented by scatters plots with bars indicating the with mean ± SEM. Student’s *t*-test, *p < 0.05. **C** Body weight (Bw) gain; **D** fasting glucose in plasma; and **E** oral glucose tolerance and area under the curve (AUC) of mice fed high-fat high-sugar diet (HFHSD) and orally administered vehicle (veh) or dead *B. uniformis* for 14 weeks. Results are represented by scatter plots indicating individual values or summary data with mean ± SEM, (*n* = 8–10 per group). Student's *t*-test: **A** and **B**. One-way ANOVA followed by Tukey’s post hoc test: **C** and **D** and AUC in **E**. Two-way ANOVA with group as between-subject factor and time as within-subject factor followed by Bonferroni’s post hoc test comparing replicate means by time: **E**. *p < 0.05 and ***p < 0.001 for comparisons indicated by the horizontal line. ^#^*p* < 0.05, ^##^*p* < 0.01 and ^###^*p* < 0.001 for HFHSD-fed groups vs CD-veh

We incubated live *B. uniformis* with human colonic epithelial (Caco-2) cells in co-culture with PBMCs, finding no changes in the abundance of naïve CD4+ T cells or Tregs (Fig. 6A). By contrast, the co-incubation of *B. uniformis* with primary cultures of naïve CD4+ T cells increased the abundance of Tregs as compared with non-stimulated cells (Fig. 6B). This suggests that structural bacterial components could reach the lamina propria to induce Treg differentiation. We thus investigated in vivo whether *B. uniformis* needs to be viable to promote its metabolic benefits by administering dead bacterial cells to HFHSD-fed wt mice (scheme of the intervention in Figure S1C). In contrast to the results with live *B. uniformis*, administration of dead bacteria failed to prevent body weight gain on HFHSD feeding (Fig. 6C). Nevertheless, administration of dead *B. uniformis* did improve fasting glycemia (Fig. 6D) and oral glucose tolerance (Fig. 6E) in diet-induced obese mice. These results suggest that structural bacterial components present in both the live and dead bacteria are sufficient to improve glucose metabolism in obesity, potentially through Treg-mediated immunosuppressive effects, but not to prevent body weight gain.

## Discussion

Our study provides core evidence on the mechanisms through which *B. uniformis,* a human intestinal bacterium isolated from a breast-fed infant, mitigates the Western diet-induced metabolic dysregulation in obesity. Specifically, we demonstrate that *B. uniformis* exerts glucoregulatory effects (but not anti-obesogenic effects) through an adaptive immunity-dependent mechanism likely triggered by motifs present in live and dead bacteria. We also found that *B. uniformis* improves nutrient sensing, thereby contributing to glucose homeostasis in obesity.

*B. uniformis* administration effectively diminished adiposity irrespective of the interactions of the bacteria with host adaptive immunity in diet-induced obesity. In additions, we conclude that the benefits of the bacterium in controlling body weight and fat mass depend on its viability, but are not mediated by the regulation of catabolic routes or ad libitum food intake. We first assessed the role of catabolic routes by investigating the fasting and refeeding responses. Fasted obese mice receiving or not *B. uniformis* showed resistance to lose weight, indicating defective fasting-related signaling responsible for the activation of catabolic routes mobilizing fat storage [33]. In contrast to our results, a previous study found that the metabolic phenotype induced by *Bacteroides acidifaciens* was attributed to the activation of adipose tissue β-oxidation and GLP-1-mediated insulin sensitivity in obese mice [34]. We also confirmed that *B. uniformis* does not reduce ad libitum caloric intake, but it could attenuate adiposity partly by limiting dietary lipid absorption, as previously described [17, 21].

We investigated how *B. uniformis* regulates metabolism in obesity, considering both endocrine and immune mechanisms. Interestingly, we show that *B. uniformis* plays a role in intestinal glucose sensing, reflected in its ability to restore the glucose-induced insulin secretion and the glucose-induced food intake suppression, both contributing to postprandial glycemic control. Indeed, this *B. uniformis* strain growing in a mucin glycan-enriched culture medium up-regulates the expression of the ortholog *clpB* gene encoding the Clp protease [18]. This is an antigen-mimetic of α-melanocyte-stimulating hormone produced by *Escherichia coli* and *Hafnia alvei* that is able to induce satiety and reduce fat mass and body weight gain [35, 36]. Accordingly, the possibility that our *B. uniformis* strain can mitigate the exacerbated food intake after fasting shown in this study and in a binge-eating model through ClpB production cannot be disregarded [37].

Further analyses of the intestinal routes where *B. uniformis* could mediate metabolic effects enabled us to disregard effects on enteroendocrine hormones involved in insulin secretion (GIP and GLP-1) and the control of food intake (GLP-1 and PYY), but support a role for *B. uniformis* in strengthening the gut barrier and the anti-inflammatory tone. In the context of obesity, *B. uniformis* seems to have a proliferative effect, enhancing the small intestine length and leading to the upregulation of *Plag2g2a* [38] and *Tlr2* gene expression. Activation of the bactericidal activity of PLAG2G2A [39, 40] and TLR2 signaling [41] might strengthen the gut barrier and counteract the increased LPS flux to systemic circulation induced by HFHSD [42], as shown here. PLAG2G2A activity could also contribute to the release of Gram-positive bacterial motifs (peptidoglycans and lipoteichoic acids), enhancing TLR2 signaling. Furthermore, *B. uniformis* increases the abundance of M2 and Tregs in effector sites such as the lamina propria, promoting an anti-inflammatory response, which limits the M1-mediated pro-inflammatory tone induced by the HFHSD, as shown in Peyer’s patches [19]. Similar to *Bacteroides fragilis*, a structural component of *B. uniformis* might promote the differentiation of functional Tregs [43, 44]. Indeed, we show that *B. uniformis* enhances Tregs in primary cultures of naïve CD4+ T cells independently of antigen-presenting cells. The molecular motif through which *B. uniformis* induces Tregs differentiation remains elusive, but previous investigations point to the polysaccharide A of *B. fragilis* as a ligand of TLR2 [43, 44].

The ability of *B. uniformis* to restore the lamina propria environment through Tregs-mediated suppression of the Western diet-associated inflammatory signals was further supported by the marked reduction in anti-inflammatory M2 cells in *Rag1*^*-/-*^ mice fed *B. uniformis*. Rather than generating proinflammatory responses to TLR ligands, M2 secrete IL10 and TGF-β 1 and, thereby, likely boost the generation of Tregs from CD4+ T cells [45]. Accordingly, *B. uniformis* might provide the microbial stimulus necessary to support the Tregs-M2 feeding loop, sustaining the restoration of the intestinal immune fitness in response to a Western diet. Nevertheless, more specific studies targeting Treg would be necessary to further confirm their key role in mediating the glucoregulatory effects of *B. uniformis* since Rag1^-/-^ mice also lack other T cells subsets and B cells.

Importantly, the present study proves that *B. uniformis* plays a major role in the adaptive immunity-mediated glucose homeostatic control, although this mechanism does not seem to be crucial to control fat storage. The action of *B. uniformis* on a functional adaptive immune system, allowing Tregs induction as we proved in vitro, might contribute to attenuate the HFHSD-induced intestinal inflammation and the disruption of the primary gut defensive mechanisms (expression of *Plag2g2a*, *Tlr2*, and *Il10* and differentiation of M2), leading to metabolic endotoxemia and glucose metabolic impairment in obesity. Nonetheless, the ability of *B. uniformis* to restore fasting hyperinsulinemia does not seem to depend on adaptive immunity. The bacterium normalized insulin levels even in a background of *Rag1-*deficiency, but this effect was not sufficient to alleviate glucose intolerance, suggesting a defect in insulin signaling caused by systemic inflammation in obesity.

Our results confirm the key role of adaptive immunity in defining the gut microbiota composition since reveal that the microbiota of *Rag1*^*-/-*^ mice is less diverse, as previously reported [46]. Of note, the microbiota configuration driven by *Rag1*-deficiency was not associated with additional metabolic derangements beyond those caused by the Western diet. In both wt and *Rag1*-deficient mice, *B. uniformis* had a minor impact on gut microbiota composition, particularly in HFHSD-fed mice, which reinforces the hypothesis that it might directly regulate adaptive immunity independently of secondary ecological effects on the intestine.

Our findings also indicate that permanent engraftment of *B. uniformis* in the distal gut is not strictly required for its immune and metabolic effects in either wt or *Rag1*^*-/-*^ mice, although we cannot rule out that the bacterium uses upper regions as ecological niches, and that this contributes to some of the effects in diet-induced obesity. We also demonstrate that live *B. uniformis* and, potentially, its metabolic by-products, are indispensable for its benefits on weight gain and adiposity but not for hyperglycemia and oral glucose intolerance.

In summary, our study identifies adaptive immunity and potentially Tregs as the leads in the cascade of immune events to recover the immune intestinal fitness with downstream benefits on blood glucose control but not on body weight and allows disregarding enteroendocrine mechanisms. It also provides new evidence on the respective role of dead and live *B. uniformis* cells in regulating glucose homeostasis or body weight and adiposity in obesity.

### Supplementary Information

**Supplementary Material 1.**

## Data Availability

All relevant data are included in the article or in the supplementary material. All datasets and raw data are available from the corresponding author on reasonable request. The fastq raw data of the 16S rRNA gene sequencing can be accessed at the European Nucleotide Archive (ENA) via bioproject number PRJEB46294.
