# Supplementary material for: Bacteroides uniformis CECT 7771 requires adaptive immunity to improve glucose tolerance but not to prevent body weight gain in diet-induced obese mice
Source: Microbiome. 2024 Jun 6;12:103. doi: 10.1186/s40168-024-01810-3 (PMC11155119; doi:10.1186/s40168-024-01810-3)
Supplement: Supplementary file 2 — Supplementary Material 1. [file 40168_2024_1810_MOESM1_ESM.docx]

**Supplementary Figure S1** Graphical representation of the *in vivo* experiments. Control diet, CD; *B.unif*, *Bacteroides uniformis* CECT 7771; high-fat high-sugar diet, HFHSD; Body weight, Bw; oral glucose tolerance test, OGTT; and wild-type, wt

**Supplementary Figure S2** (related to Figure 1) (A) weekly body weight (Bw) evolution along 14 weeks of the experiment; (B) weight of the inguinal white adipose tissue (iWAT) after 14 weeks of high-fat high-sugar diet (HFHSD)/control diet (CD)-feeding; (C) GLP-1 and (D) GIP plasma levels after 12 weeks of HFHSD/CD-feeding in fasting and 10 min after an oral glucose load; (E) individual caloric intake after 13 weeks of HFHSD/CD-feeding; (F) caloric intake at 3, 6 and 24 h of refeeding at week 6 of the experiment (G) PYY plasma levels in fasting and after an oral glucose load after 12 weeks of HFHSD/CD-feeding. Results are represented by dot plots indicating individual values or summary data with mean ± SEM, (n = 5–10 per group). Two-way ANOVA with group as between-subject factor and time as within-subject factor followed by Bonferroni’s post hoc test comparing replicate means by time: (A) and (F). Kruskal-Wallis followed by Dunn’s multiple comparison test: (B) and (E). Paired t tests: (C), (D) and (G) with time as within-subject factor. **p < 0.01 for HFHSD-veh vs HFHSD-*B.unif* comparisons. ^#^p < 0.05, ^##^p < 0.01 and ^###^p < 0.001 for HFHSD-fed groups vs CD-veh group.

**Supplementary Figure S3** (related to Figure 2) (A) heatmap representing the fold-change gene expression relative to control diet (CD)-fed mice receiving vehicle (CD-veh) of *glut2* and *sgtl1* in duodenum; (B) heatmap representing the fold-change gene expression relative to CD-veh of *defA*, *reg3ɤ*, *lyz1*, *plag2g2a* and *muc2* in ileum; (C) heatmap representing the fold-change gene expression relative to CD-veh of *nod1*, *nod2*, *tlr2*, *tlr4*, *tlr5*, *tnfα*, *il10* in ileum, and (D) percentage of natural and induced intraepithelial cells (natIEL and indIEL, respectively) in the epithelium of the small intestine of mice fed HFHSD and receiving either vehicle (veh) or *B.uniformis* (*B.unif*), or of CD-veh mice. Results are represented by dot plots with mean ± SEM or heatmap representing the gene expression fold-change relative to CD-veh, (n = 7–10 per group). One-way ANOVA followed by Tukey’s post hoc test: *reg3ɤ*, *plag2g2a* and *muc2* in (B); *tlr2*, *tlr5*, *tnfα* and *il10* in (C); and natIEL in (D); Kruskal-Wallis test followed by Dunn’s multiple comparison test: *glut2, sgtl1* in (A); *defA and lyz1* in (B); *nod1*, *nod2* and *tlr4* in (C); and indIEL in (D); Mann-Whitney U test for HFHSD-fed groups: indIEL in (D); Pearson correlation: (E). ^##^p < 0.01 and ^###^p < 0.001 for HFHSD-fed groups vs CD-veh group.

**Supplementary Figure S4** (related to Figure 3) *Rag1*-deficient (*Rag1^-/-^*) or wild-type (wt) mice orally receiving vehicle were used to identify potential confounding effects of the genotype on the metabolic phenotype. Exploration of the effects of the diet, the genotype, and interactions between both variables on (A) body weight (Bw) gain; weight of (B) epididymal and (C) inguinal white adipose tissue (eWAT and iWAT) and (D) brown adipose tissue (BAT) and (E) liver; (F) length of the small intestine; and (G) oral glucose tolerance test and the area under the curve (AUC) in *Rag1^-/-^* and wt mice fed HFHSD or CD for 14 weeks. Results are represented by dot plots indicating individual values or summary data with mean ± SEM (n = 10 per group). Two-way ANOVA with genotype (*Rag1^+/+^* or wt) and diet (CD or HFHSD) as between-subject factors: (A–F) and AUC in (G). Two-way ANOVA with group as between-subject factor and time as within-subject factor followed by Bonferroni’s post hoc test comparing replicate means by time: (G). ^#^p < 0.05, ^##^p < 0.01 and ^###^p < 0.001 for HFHSD vs CD in either *Rag1^-/-^* or wt mice

**Supplementary Figure S5** (related to Figure 3) Insulin levels in plasma in *Rag1*-deficient mice (*Rag1^-/-^*) fed control diet (CD) or high fat high sugar diet (HFHSD) and orally receiving vehicle or *B. uniformis* for 14 weeks. Results are represented by dot plots indicating individual values with mean ± SEM (n = 10 per group). Two-way ANOVA with diet (CD or HFHSD) and treatment (vehicle or *B. uniformis*) as between-subject factors followed by Tukey’s *post hoc* test. ***p<0.001 for comparisons indicated by the horizontal line. ^###^p < 0.001 for HFHSD-fed groups vs CD-veh in either *Rag1^-/-^* or wt mice.

**Supplementary Figure S6** (related to Figure 5) Fecal samples from mice receiving vehicle were used to determine interactions between the diet (CD or HFHSD) and genotype (wt or *Rag1^-/-^*) in the alpha and beta diversity analyses and bacterial taxonomy. (A) Chao’s richness, Simpson’s reciprocal index, and phylogenetic distance (PD) estimators. Comparison between groups were carried out through the Wilcoxon Rank Sum test after the Shapiro-Wilk normality test. Benjamini--Hochberg post hoc correction when analyzing genotype × diet interactions was applied after multiple group pairwise comparisons (p-adj). Data distributions are shown in boxplots. Color legend is shown above panel (A) and medians for all groups are represented by dotted grey lines projected from y-axis. (B) Constrained distance-based (Bray-Curtis) redundant analysis (db-RDA). Results of Adonis test of group comparisons is shown within the scatter plot as well as the group labels. (C) Bacterial taxonomy groups altered in different mouse groups. Comparison among groups is supported by application of pairwise Wilcoxon Rank Sum test. P-values on top of respective panels correspond to adjusted p-values following *post hoc* correction (Benjamini-Hochberg). Color legend is shown at the bottom of the figure and medians are represented by dotted grey lines projected from y-axis.

**Supplementary Table S1:** genes and primer pair sequences

| **Gene name** | **Abbreviation** | **Sequence 5´- 3´** | **Supplier** |
| --- | --- | --- | --- |
| Defensin A | ***DefA*** | Forward: GGTGATCATCAGACCCCAGCATCAGT  Reverse: AAGAGACTAAAACTGAGGAGCAGC | Isogen Life Science |
| Glucose transporter 2 | ***Sclc2a2*** | Forward: TTGTGCTGCTGGATAAATTC  Reverse: AAATTCAGCAACCATGAACC | Sigma-Aldrich |
| Interleukin 10 | ***Il10*** | Forward: GCTCTTACTGACTGGCATGAG  Reverse: CGCAGCTCTAGGAGCATGTG | Isogen Life Science |
| Lysozyme 1 | ***Lyz1*** | Forward: GCCAAGGTCTACAATCGTTGTGAGTT  Reverse: CAGTCAGCCAGCTTGACACCACG | Isogen Life Science |
| Mucin 2 | ***Muc2*** | Forward: CCCAGAAGGGACTGTGTATG  Reverse: TGCAGACACACTGCTCACA | Isogen Life Science |
| Nucleotide-binding oligomerization domain-containing protein 1 | ***Nod1*** | Forward: TCCCTTGCCTGTGAGCAGAAAGTA  Reverse: GTGGGTATGTGCCATGCTTTGCTT | Isogen Life Science |
| Nucleotide-binding oligomerization domain-containing protein 2 | ***Nod2*** | Forward: CACACATGGCCTTTGGTTTCCAGT  Reverse: AAAGAGCTGCAGTTGAGGGAGGAA | Isogen Life Science |
| Phospholipase A2 group IIA | ***Pla2g2a*** | Forward: AAGGATCCCCCAAGGATGCCAC  Reverse: CAGCCGTTTCTGACAGTTCTGG | Isogen Life Science |
| Regenerating islet-derived protein 3 gamma | ***Reg3ɤ*** | Forward: TTCCTGTCCTCCATGATCAAA  Reverse: CATCCACCTCTGTTGGGTTC | Isogen Life Science |
| Ribosomal protein L19 | ***Rpl19*** | Forward: CCTTGTCTGCCTTCAGCTTGT  Reverse: GAAGGTCAAAGGGAATGTGTTCA | Isogen Life Science |
| Sodium-glucose linked transporter 1 | ***Slc5a1*** | Forward: GCCCTTTATACAATCACAGG  Reverse: AAGGCTTCATATCCTCCTAC | Isogen Life Science |
| Toll-like receptor 2 | ***Tlr2*** | Forward: CAACTTACCGAAACCTCAGACAAAG  Reverse: GCCACCAAGATCCAGAAGAGC | Isogen Life Science |
| Toll-like receptor 4 | ***Tlr4*** | Forward: GCCCAGGCTTTTGTCAAACA  Reverse: CTCCCCACACACCAGGTAGAG | Isogen Life Science |
| Toll-like receptor 5 | ***Tlr5*** | Forward: CTTCCCTGGAGTCATTTTTC  Reverse: AGACAGTACGCAATAGGATG | Sigma-Aldrich |
| Tumor necrosis factor alpha | ***Tnfα*** | Forward: TGTCTCAGCCTCTTCTCATTCC  Reverse: TGAAAATCTGGGCCATAGAAC | Isogen Life Science |

**Supplementary Table S2:** Antibodies used for the analysis of immune cells in the small intestine by flow cytometry

| **Immune cells** | **markers** | **antibodies** | **Supplier** |
| --- | --- | --- | --- |
| **Intraepithelial lymphocytes** | Natural: CD45+ CD2+ CD5+  Induced: CD45+ CD2- CD5- | FITC-conjugated anti-CD45 | Miltenyi Biotec (Germany) |
|  |  | PE-Vio770-conjugated anti-CD2 | Miltenyi Biotec (Germany) |
|  |  | APC- conjugated anti-CD5 | Miltenyi Biotec (Germany) |
| **Macrophages** | M1: F4/80+ CD80+ iNOS+  M2: F4/80+ CD206+ Arg1+ | FITC-conjugated anti-F4/80+ | Miltenyi Biotec (Germany) |
|  |  | Pe-Vio770-conjugated anti-CD80 | Miltenyi Biotec (Germany) |
|  |  | APC-conjugated anti-iNOS* | Thermo Fisher Scientific (USA) |
|  |  | PerCPCy5.5-conjugated anti-CD206 | Biolegend (USA) |
|  |  | PE-conjugated anti-Arg1* | R&D Systems (USA) |
| **T cells** | CD4+ T CD25- Foxp3-  Treg: CD4+ CD25+ Foxp3+ | BV711-conjugated anti-CD4 | BD-Bioscience (USA) |
|  |  | APCCy5.5-conjugated anti-CD25 | BD-Bioscience (USA) |
|  |  | PE-conjugated anti-Foxp3* | Miltenyi Biotec (Germany) |
| *antibodies against intracellular markers | | | |
